# Establishing a case for improved food phenolic analysis

**DOI:** 10.1002/fsn3.74

**Published:** 2013-10-31

**Authors:** Jungmin Lee

**Affiliations:** Horticultural Crops Research Unit Worksite Agricultural Research Service, United States Department of AgricultureParma, Idaho, 83660, USA

**Keywords:** Phenolics, polyphenolics, quality, secondary metabolites

## Abstract

Future phenolic research findings using a multidisciplinary approach will ensure profitability and sustainability of specialty crop industries, while also improving the nutritional and economic choices available to increasingly health- and environmentally conscious consumers. Recent examples of phenolics used in commercial and research scenarios, and new phenolic research discoveries are discussed. Despite being a heavily researched topic, there remains a need to identify, develop, and define analyses targeted for specific quality-related plant metabolites.

## Introduction

Improved consumer awareness about healthy food choices has resulted in an increase in market demand and consumption of small fruits, herbs, tree nuts, and vegetables. Although a number of market segments are undergoing revitalized product diversity, the majority of the cases cited in this review are either fruit or fruit products (except one example). For example, the edible desirability of fruits stems from their primary and secondary metabolites that contribute toward fruit quality. Phenolics make up a small portion of the compounds present in a fruit or its final product, but they are crucial for their contribution to appearance (color, haze), taste (bitterness and astringency), storability, and potential health benefits (Cheynier 2005; Lee et al. 2012; Tomas-Barberan and Andres-Lacueva 2012). Plant phenolics are a diverse group of plant secondary metabolites with over 6000 identified (Maeda and Dudareva 2012). Phenolics remain a heavily researched topic due to their roles within plants, and importance to consumers. These compounds are implicated in having several specific plant functions, including ultraviolet (UV) radiation protection, pigmentation, antifungal/antimicrobial properties, hormonal signaling, attraction/repulsion of pollinators and seed dispersers, and nodule production (Agati et al. 2012).

**Figure 1:**
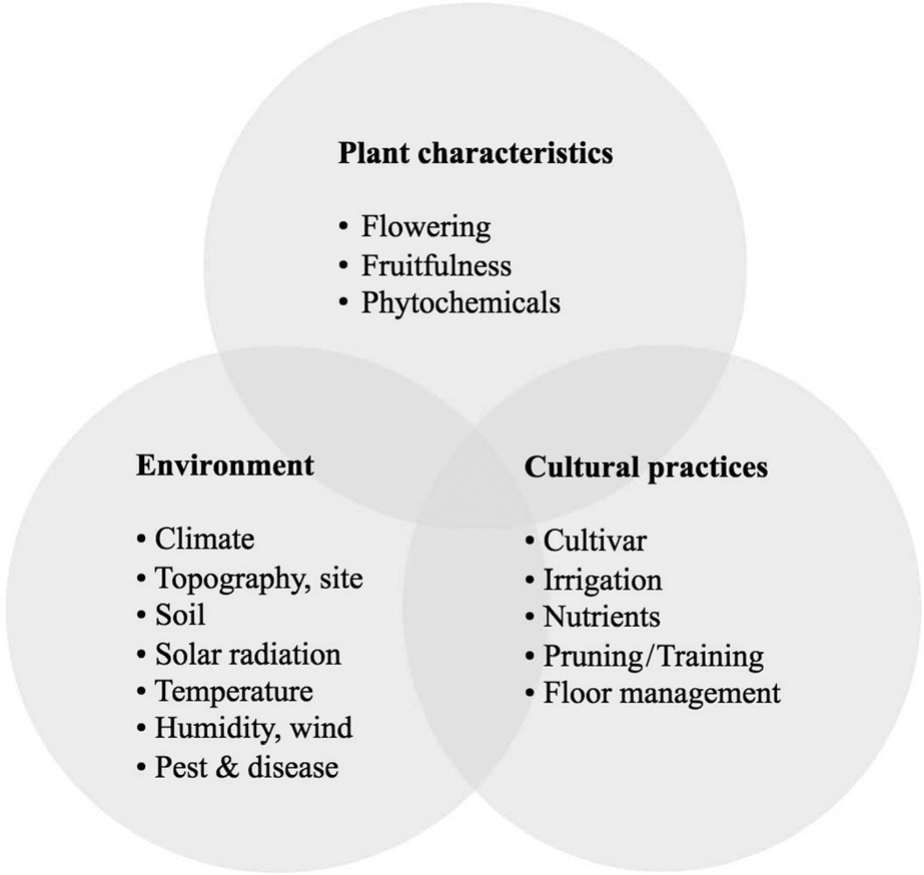
A list of biotic and abiotic factors that can influence fruit phenolics prior to harvest, processing, sample preparation, and analysis.

Many variables (Fig. 1) affect the ultimate phenolic content of fruit and fruit products, including horticultural, genetic, environmental, and processing factors (Lee et al. 2002, 2004a,2004b, 2008b, 2012; Lee and Wrolstad 2004; Lee and Finn 2007; Tarara et al. 2008; Lee and Martin 2009; Lee 2010a; Lee and Skinkis 2013; Lee and Steenwerth 2013; Mosse et al. 2013; Schreiner et al. 2013; Thornton et al. 2013). The study of phenolics is very complex as other compounds (e.g., free amino acids, carbohydrates, organic acids) involved in plant metabolite biosynthesis also contribute to quality appearance and flavor parameters, and affect fermentation and processing behavior. Significant additional research is needed to fully understand the role of phenolics in potential human health benefits (Tomas-Barberan and Andres-Lacueva 2012), but that area is not within the scope of this review.

Phenolics can be divided into two groups based on their structure: nonflavonoids (phenolic acids, stilbenes, and ellagitannins) and flavonoids (anthocyanins, flavonol-glycosides, flavanol monomers, and proanthocyanidins), and both have been well reviewed regarding its distribution in plant, resulting food, and structural elucidation (Cheynier 2005; Arapitas 2012; Lee et al. 2012). Recent incidents of product adulteration or mislabeling, when genuine phenolic-containing fruit juices and concentrates command a high price premium, highlight their consumer regard and need for specific testing (Penman et al. 2006; Lee 2013; Primetta et al. 2013). A 2008 import wine scandal, where wine labeled and sold as French “Pinot noir” in the United States was actually a blend of “Merlot” and “Syrah” wine (final settlement approved in 2012), emphasizes the importance of widening our phenolic profile databases (e.g., Neveu et al. 2010; http://www.phenol-explorer.eu) for fruit phenolic authentication. More accurate fruit phenolic identification requires reliable and effective analytical methods. The objective of this review was to provide a brief overview of the importance, measurements, and applications of phenolics by providing actual examples and recent research findings.

### Phenolic measurements and challenges

While the need for better identification of phenolic monomers is widely recognized, the predominant fruit phenolics are phenolic polymers (i.e., proanthocyanidin-condensed tannins and ellagitannin-hydrolyzable tannins), for which there is very little literature. This is a much-needed area of research that will aid investigations in many fields. Among them are the roles phenolic polymers play in plant development (Salminen and Karonen 2011), and phenolic analyses of ellagitannin evolution in wine (introduced by wood; Versari et al. 2013). Polymers present a complex puzzle to decipher as they are extremely challenging to isolate, purify, and analyze (Lee et al. 2008b, 2012; Koerner et al. 2009; Lee 2010a, 2013; Lee and Rennaker 2011; Arapitas 2012). Despite extreme care, even routine handling of samples prior to analysis (e.g., freezing, gentle extraction, or purification steps) can alter native polymer structures and degrade or break down the compounds under observation (Hakkinen et al. 2000; Hager et al. 2008; Gasperotti et al. 2010; Salminen and Karonen 2011). Because their structural heterogeneity has made them frequently overlooked, few methods or standard reference materials are available (Vrhovsek et al. 2006, 2008; Gasperotti et al. 2010; Salminen and Karonen 2011; Arapitas 2012; Lee et al. 2012). The gaps in quantitative hydrolysis and analysis of the phenolic polymers data need to be filled, while method performance characteristics have to be defined for researchers unfamiliar with phenolic analyses.

There are numerous phenolic extraction and analysis techniques reported in the literature (Wrolstad et al. 2005; Lee et al. 2012) but very few properly validated methods are available. Validated methods are important as they allow comparison among laboratories (Lee et al. 2005b; Brooks et al. 2013). The values obtained by using validated methods have known reproducibility, repeatability, and defensibility (Lee et al. 2005b; Brooks et al. 2013); the techniques have clearly evaluated and defined performance characteristics (Lee et al. 2005b, 2012; Brooks et al. 2013). Despite the difficulty and cost in validating methods, the importance of well-defined approaches has been highlighted during recent controversies regarding condensed tannin analyses (Brooks et al. 2008), anthocyanin analyses (Brooks et al. 2013), and in vitro antioxidant analyses (Gilsenan 2011; Agati et al. 2012; Berger et al. 2012; Tomas-Barberan and Andres-Lacueva 2012; Chiva-Blanch and Visioli 2013). Recent problems with tannin analyses (Harbertson et al. 2003) stemmed from a lack of reproducibility and repeatability, as an independent interlaboratory review demonstrated (Brooks et al. 2008). Many U.S. wineries were utilizing this nonvalidated tannin method as part of their winemaking decisions (e.g., duration of skin and seed contact time), only to later realize numerous method restrictions existed (Brooks et al. 2008; Jensen et al. 2008) and that interlaboratory study-obtained values varied widely (Brooks et al. 2008). Some corporations provide grower bonuses based on harvested fruit color content (Lee 2013). The importance of fully developed and validated methods, in the case of phenolics, can be directly linked to profit (Lee 2013), from value-grading produce to marketing claims for consumer attention (Mercurio et al. 2010; Lalor et al. 2011; Caceres et al. 2012; Lawless et al. 2012). However, some commercial product manufacturers make claims that are not true. For example, although they allege high phenolic levels, cranberry supplements per dose contained less proanthocyanidins than a serving of cranberry juice cocktail (Lee 2013).

Increased availability and accessibility of mass spectrometry (MS) has led some researchers (Wu and Prior 2005; Seeram et al. 2006; Wu et al. 2006; Prior et al. 2009; Cuevas-Rodriguez et al. 2010; Kellogg et al. 2010) in misidentifying and simply missing main peaks for phenolic identification by not utilizing co-chromatography of well-established plant materials, peak UV–visible (UV–Vis) spectra comparison, retention time, etc. The current plant phenolic analysis trend is untargeted and targeted metabolomic analyses (Wishart 2008; Patti 2011; Vrhovsek et al. 2012). Progress is hampered by the high cost of acquiring, operating, and maintaining the instrumentation needed for those techniques (i.e., high-resolution mass spectrometers or nuclear magnetic resonance spectrometers), and by the lack of well-established or standard procedures and compound identifications (Scalbert et al. 2009; Arapitas 2012; Lee et al. 2012). While there are analytical uses for untargeted and targeted plant metabolomics, the technologies have limitations (well reviewed by Wishart 2008; Scalbert et al. 2009), and traditional phenolic research techniques of grouped-by-similar-structural classes remain valuable. There is a need for well-defined, specifically targeted analytical methods for analyses for phenolics in small fruits; similar to methods that have been developed in the past (Lee and Harnly 2005; Lee et al. 2005a,2005b, 2008a,2008b, 2009; Lee and Finn 2007; Koerner et al. 2009; Lee and Scagel 2009, 2010; Lee 2010a, 2013; Lee and Schreiner 2010; Lee and Rennaker 2011). Systematic phenolic identification procedures that only require customary tools, alongside modern contemporary methods, should be considered part of any phenolic research strategy to improve the accuracy and reliability of metabolite identification.

### Examples demonstrating phenolic research application and opportunities

**Figure 2:**
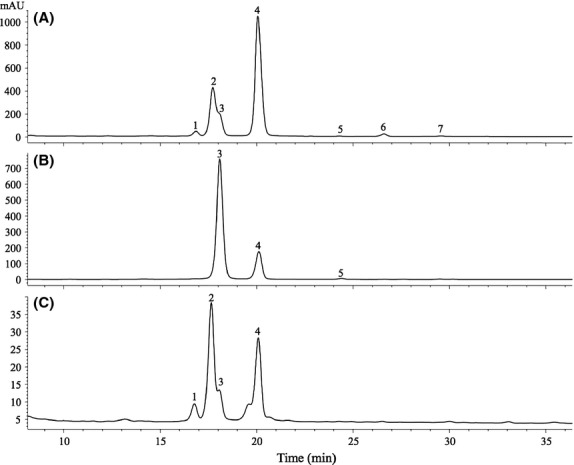
Anthocyanin profiles of *Rubus occidentalis* “Munger” fruit (A), *Rubus coreanus* fruit (B; also referred to as bokbunja), and commercial juice sample labeled as bokbunja (C) monitored at 520 nm. Additional information to aid distinguishing these two species can be found in Lee et al. (2013). Peaks: 1, cyanidin-3-sambubioside; 2, cyanidin-3-xylosylrutinoside; 3, cyanidin-3-glucoside; 4, cyanidin-3-rutinoside; 5, pelargonidin-3-glucoside; 6, pelargonidin-3-rutinoside; 7, peonidin-3-rutinoside. Clearly, *R. coreanus* fruit (B) lacks peaks 1, 2, 6, and 7. The unique anthocyanin profile can be used for food authenticity work. A sample of commercially available bokbunja juice (C) labeled as containing 7% bokbunja from concentrate; however, based on its anthocyanin profile it unmistakably contained juice from *R. occidentalis* fruit, not *R. coreanus* fruit. Juice sample was prepared and analyzed as described in Lee and Finn (2007) and Lee (2013).

Although there is a large body of research on phenolics, there are still opportunities to make new discoveries and solve disparities among results, four examples are provided from our research efforts:

1. The second major basil (*Ocimum basilicum* L.) leaf phenolic was recently identified as chicoric acid that has similar UV–Vis spectra and a mass fragmentation pattern to grapes' main phenolic acid, caftaric acid (Lee and Scagel 2009, 2010; Lee 2010b; Scagel and Lee 2012). Chicoric acid easily and rapidly degrades during customary extraction procedures and processing (Stuart and Wills 2003; Lee and Scagel 2009; Lee 2010b), and we suspect this was one reason the identification was overlooked for so long. The importance of the sample extraction step for high-phenolic retention was demonstrated by introducing a straightforward blanching step (Lee and Scagel 2009). This line of investigation can also directly improve commercial processing as well (Lee 2010b), as we found that even basil prepared by freeze-drying (gentler drying than the ordinary open air process) contained 78% less phenolics than it had at peak concentration (Lee 2010b). Sample preparation is often neglected in quality analysis research, although it is the critical first chemical analysis step that can affect results (Kim and Verpoorte 2010; Lee and Schreiner 2010; Lee and Rennaker 2011; Lee et al. 2012). Continued refinements of sample handling, preparation, hydrolysis, and purification steps that optimize phenolic retention will advance research evaluations and production processes.
2. Identity of the major black raspberry (*Rubus occidentalis* L.) anthocyanin was clarified (Dossett et al. 2008, 2010, 2011; Lee et al. 2012). Others (Wu and Prior 2005; Seeram et al. 2006; Wu et al. 2006; Prior et al. 2009) had previously relied on MS results and incorrectly identified cyanidin-3-xylosylrutinoside as cyanidin-3-sambubioside-5-rhamnoside. Adding to the confusion, these incorrectly identified black raspberry anthocyanins were unintentionally then used for in vitro and in vivo studies in the hope of better understanding their pharmacokinetic mechanisms (Seeram et al. 2006; Wu et al. 2006; Prior et al. 2009). However, without correct identifications, phenolic consumption-tracking findings become questionable. Researchers conducting animal and human studies on the benefits of phenolics, which inadvertently rely on inaccurate work, could be tracing unintended compound metabolic pathways. This emphasizes the importance of correct identification of the starting material for the benefit of other scientific studies downstream.
3. A wild black raspberry genotype bearing fruit with a unique anthocyanin profile was discovered (Dossett et al. 2011). The plant was part of a wild-germplasm collection that was grown in a research plot as part of investigations on genetic diversity for breeding improved black raspberry cultivars. Fruit of this genotype lacked anthocyanins containing rutinosides. Their distinctive profile provides an opportunity to study the genetic control over that portion of the anthocyanin biosynthetic pathway. Even in the widely studied *Rubus* fruit, opportunities remain for discovering new anthocyanin profiles. Continued work to develop and deploy analytical methods in characterizing unique plant phenolic profiles for improving fruit quality that can be used in guiding breeding programs, and identifying adulteration in food investigations is needed.
4. The confusion surrounding the identity of Korean black raspberry (bokbunja; *Rubus coreanus* Miq.) plant, fruit, and anthocyanin profile was clarified (Lee et al. 2013; see Fig. 2). Most Korean black raspberry growers are unknowingly growing *R. occidentalis* L. (not bokbunja), as demonstrated by Lee et al. (2013) that the pigment fingerprint is unique for each of the two species. This allows its use as a taxonomy criterion for food authenticity/adulteration work (see Fig. 2). Bokbunja fruit contained the following anthocyanins: cyanidin-3-glucoside, cyanidin-3-rutinoside, and pelargonidin-3-glucoside, with pelargonidin-3-glucoside not detected in all bokbunja samples (Lee et al. 2013). *Rubus occidentalis* L. fruit contained additional cyanidin-3-sambubioside, cyanidin-3-xylosylrutinoside, pelargonidin-3-rutinoside, and peonidin-3-rutinoside besides the three anthocyanins found in bokbunja fruit, and pelargonidin-3-rutinoside and peonidin-3-rutinoside are not detected in all *R. occidentalis* L. fruit (Dossett et al. 2010; Lee et al. 2012). Due to identity mix-ups of bokbunja, research claiming to be conducted on bokbunja fruit (see Table 1) requires confirmation that the fruit was sourced from a correctly identified plant. A list of recent research conducted on correctly and incorrectly identified *R. coreanus* fruit are listed in Table 1. Consumers and producers who value true bokbunja for its traditional cultural significance will benefit from this work.

**Table 1:** List of recent research papers on correctly and incorrectly identified *Rubus coreanus* fruit based on anthocyanin profile shown in Figure 2.

Title of manuscript	Correct fruit studied?	Citation
Metabolite fingerprinting of bokbunja (*R. coreanus* Miquel) by UPLC-qTOF-MS	Yes	Heo et al. (2011)
Antiulcer activity of anthocyanins from *R. coreanus* via association with regulation of the activity of matrix metalloproteinase-2	Yes	Kim et al. (2011a)
Anthocyanins in the ripe fruits of *R. coreanus* Miquel and their protective effect on neuronal PC-12 cells	No	Im et al. (2013)
Biochemical monitoring of black raspberry (*R. coreanus* Miquel) fruits according to maturation stage by ^1^H NMR using multiple solvent systems	No	Kim et al. (2011b)
Optimization of the extraction of anthocyanin from Bokbunja (*R. coreanus* Miq.) marc produced during traditional wine processing and characterization of the extracts	No	Ku and Mun (2008)
Protective actions of *R. coreanus* ethanol extract on collagenous extracellular matrix in ultraviolet-B irradiation-induced human dermal fibroblasts	No	Bae et al. (2007)

## Concluding Remarks

Long before fruit or fruit products enter the laboratory for phytochemical extraction and analysis, their phenolic profiles and concentrations are dependent upon numerous variables. Solar radiation, temperature, virus status, and other biotic and abiotic stresses affect phenolic content (Lee et al. 2008a; Tarara et al. 2008; Lee and Martin 2009; Lee and Schreiner 2010; Remberg et al. 2010; Lee and Skinkis 2013). Increasing our comprehension of the roles phenolics play in plants involves a multidisciplinary research approach and well-defined relationships of links among fruit metabolites, agricultural factors, and desired fruit attributes. Continued effort to decipher those links that increase phenolic retention in products reaching consumers is needed, and these links may lead to further investigation opportunities.
